# A Heterogeneity Study of Carbon Emissions Driving Factors in Beijing-Tianjin-Hebei Region, China, Based on PGTWR Model

**DOI:** 10.3390/ijerph19116644

**Published:** 2022-05-29

**Authors:** Ting Lou, Jianhui Ma, Yu Liu, Lei Yu, Zhaopeng Guo, Yan He

**Affiliations:** 1 School of Economics, Hebei University, Baoding 071002, China; louting15103313558@126.com (T.L.); liuyu13910108397@163.com (Y.L.); 18734829093@163.com (L.Y.); guozp_student@126.com (Z.G.); heyan17366522806@163.com (Y.H.); 2 Research Center of Resources Utilization and Environmental Conservation, Hebei University, Baoding 071002, China; 3 School of Management and Economics, Tianjin University, Tianjin 300072, China

**Keywords:** carbon emission, Beijing–Tianjin–Hebei region, PGTWR model, heterogeneity, driving factors, city, econometrics

## Abstract

The Beijing–Tianjin–Hebei region is an important economic growth pole in China and achieving carbon emission reduction in the region is of great practical significance. Studying the heterogeneity of the influencing factors of carbon emission in this region contributes to formulating targeted regional carbon emission reduction policies. Therefore, this paper adopted thirteen cities as individuals of cross-section and conducted spatial and temporal heterogeneity analysis of the influencing factors of converted carbon emissions in the region with panel data from 2013 to 2018 based on the PGTWR model. From a space-time perspective, the regression coefficient of each influencing factor in this region has obvious heterogeneity, which is mainly reflected in the time dimension. In the study period, the impact of industrial structure, the level of urbanization, energy intensity, and the level of economic growth on carbon emission showed a decline curve, while the impact of the level of opening up and the size of population was on the rise, indicating that more attention should be paid to the latter two factors for the time to come. In terms of space, the differences in the influence of industrial structure and energy intensity on carbon emission vary significantly.

## 1. Introduction

Global warming has become a serious concern to all countries in the world. Special Report on Global Warming of 1.5 °C, issued on IPCC, pointed out that the space of global carbon emission has been very limited and that it is quite urgent to cope with climate change and achieve the goal of the temperature control of 1.5 °C or 2 °C [1]. Emissions Gap Report 2020, issued on UNEP, indicated that as the largest carbon emitter, China’s greenhouse gas emissions accounted for 26.7% of the global total in 2019. Hence, great emphasis should be laid on carbon emission reduction. On 22 September 2020, President Xi Jinping stated for the first time that China will “strive to achieve carbon peaking by 2030 and carbon neutrality by 2060”. 

As a significant economic growth pole in northern China and a region with a high concentration of high-carbon-emission industries represented by the steel and petrochemical industry, the Beijing–Tianjin–Hebei metropolitan Economic Circle withstands enormous pressure of the regional carbon emission reduction. Xi stressed that the cooperative development of the Beijing–Tianjin–Hebei region, which serves as a major national strategy of China, should persist in mutual benefit and solid advancement while accelerating in finding a scientific and sustainable path of coordinated development. Carbon emission and economic development are a complex and comprehensive system. Regional coordinated development should not only realize coordinated economic development, but also consider ecological environment and attain regional carbon emission reduction. The influencing factors of carbon emissions usually possess geographical-temporal heterogeneity [2,3,4,5]. Geographically, there are differences in economic development, industrial structure, and other factors among regions. Temporally, there are also inevitably differences in the degree of development of regions in the past and at present. Therefore, when we investigate carbon emissions driving factors, its temporal and spatial heterogeneity should be fully considered to obtain a more accurate result. Heterogeneity studies on influencing factors of carbon emissions in China are mainly focused on eastern developed regions such as Shanghai and Zhejiang, and little attention is paid to the Beijing–Tianjin–Hebei region. Hence, in-depth exploration of driving factors of carbon emissions in the Beijing–Tianjin–Hebei region from the perspective of the spatial and temporal heterogeneity, accurate identification of the direction and degree of influence of each driving factor on carbon emissions in different times and space, and provision of carbon emission targeted strategies of differentiation and inter-district joint governance is of great realistic significance to the realization of the goals of carbon peaking and carbon emission reduction at a regional and national scale.

At present, domestic and foreign scholars’ research on the influencing factors of carbon emissions was carried out based on four types of quantitative methods. The first is based on the factor decomposition method [6,7,8]. The second type is the STIRPAT model-based research [9,10]. Most of the above research are the global econometric models based on time series data without considering the spatial effect among variables, namely geographic dependence and geographic heterogeneity. From the perspective of the former, scholars have carried out the third category of studies, which adds spatial lag variables or lag error terms into the model. The models adopted mainly encompass the spatial error model (SEM), spatial lag model (SLM), and spatial Durbin model (SDM) [11,12], which still fall into global econometric models. The fourth type of research, from the perspective of the heterogeneity, uses the Geographically Weighted Regression (GWR) model, Geographically and Temporally Weighted Regression (GTWR) model, and Panel Geographically and Temporally Weighted Regression (PGTWR) model to describe the differences of the influence coefficients of these driving factors in different time and space, thus reflecting the reality more scientifically, realistically and accurately [2,3]. The research in this paper also belongs to the fourth category in which more detailed literature review is conducted.

The earliest model which was used to analyze the spatial heterogeneity of variable relations is GWR model. Brunsdon et al. and Pavlov [13,14] initially analyzed the influence of spatial heterogeneity of various factors on housing price and most of the current GWR model-based applications also fall into the same category [15,16]. In addition, GWR model is widely used in the following research including regional economic development [17,18,19], spatial difference of Income Distribution and Influence Factors [20,21], spatial pattern of regional innovation [22,23], urban economy and ecology [24,25], ecological environment [26,27], and spatial heterogeneity of carbon emissions [27,28]. The disadvantage of the GWR model is that it is only applicable to large-sample cross-sectional data and can only analyze geographic heterogeneity and fail to reflect temporal heterogeneity of variable relationships. In view of the defects of GWR, Huang et al. [29] extended GWR model to GTWR model by embedding the time factor into the spatial weight matrix, which is based on the Gaussian kernel function and Euclidean distance while comparing the regression results of Temporally Weighted Regression (TWR) model. Thus, the conclusion that GTWR was better was drawn.

After that, GTWR has been widely used in research on temporal and spatial heterogeneity, e.g., influencing factors of housing price [29,30], influencing factors of provincial economic development [31], temporal and spatial characteristics of hydrology [32,33], atmospheric pollutant emission driving factors [34,35,36], and driving forces of urban expansion [37]. Some scholars analyzed the temporal and spatial heterogeneity of different influencing factors of carbon emissions by means of the GTWR model [2,3]. However, the GTWR model only did a cross-section processing of panel data, which does not meet the need for local analysis and modelling of panel data. Meanwhile, these models also ignore the indirect path of the mapping process from the information of the sample region to the target analysis region and also ignore the temporal transfer and conducting effect of the spatial spillover effect of the sample region. 

Given the defects of the GTWR model, Fan and Guo [38] proposed the holographic mapping-based approach, which structures the unified framework and analytical paradigm of the panel geographic-temporal weighted regression model applicable to the local analysis of panel data space. The model not only reflects the characteristics of the panel data model, but also comprehensively analyzes the direct path and indirect path of influence between local points in space. Thus, the GTWR model is fundamentally improved by including optimal spatial bandwidth and optimal temporal bandwidth into the effective nearest neighbor local points, which analyzes regularity and heterogeneity of spatial dependence of local points more accurately.

The paper starts off studying the gap and focuses on using the PGTWR model to analyze the temporal and spatial heterogeneity of the influencing factors of carbon emissions in the Beijing–Tianjin–Hebei region. The objective of our research is to propose targeted policy recommendations for low-carbon transformation development and attainment of carbon neutrality in this region.

The paper mainly answers the following research questions:

i. Which of the eight model estimation results of the PGWTR model reflects superior overall statistics properties?
ii. Degree and characteristics of spatial and temporal heterogeneity of carbon emissions in the Beijing–Tianjin–Hebei region
iii. How do governments at all levels in the region formulate carbon emission reduction policies?

The structure of the paper is as follows: The second chapter is an introduction of the process of model construction and data source. The third one is about the model numerical results and analysis of the spatial and temporal heterogeneity. The fourth one is conclusions and policy recommendations.

## 2. Materials and Methods

### 2.1. PGTWR Model

**Figure 1:**
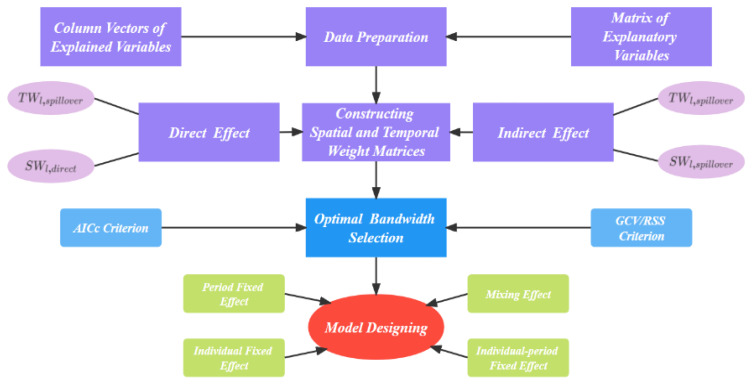
An overview of the PGTWR model.

This article adopted the PGTWR model proposed by Fan and Guo [38] as benchmark model. It is a local linear regression model based on the concept of holographic mapping, which reflects the characteristics of panel data model and can simultaneously consider the all-round and spatially-temporally dimensional effect of neighboring and local points on the target analysis region. An overview of the PGTWR model is showed below (Figure 1). Its basic model is displayed in Equation (1):

(1) $$y_{\left\{i,t\right\}}=\beta_{0}\left(i,t\right)+\beta_{1}\left(i,t\right)X_{1,\left\{i,t\right\}}+\beta_{2}\left(i,t\right)X_{2,\left\{i,t\right\}}+\cdots +\beta_{k}\left(i,t\right)X_{k,\left\{i,t\right\}}+\mu_{\left\{i,t\right\}}$$

*y* denotes the explained variable, *X* represents the explanatory variable, *β* denotes the regression coefficient, i denotes individuals of cross-section, t denotes time, and μ denotes stochastic disturbance term to satisfy the classical assumption.

The modeling steps are as follows:

1. Select samples, arrange the data according to certain rules, thus forming the matrix of variable explained and of explanatory variable.

2. Based on the location information of the sample region’s time–space dimension and by means of the kernel function, the paper converted the information of the sample region’s temporal and spatial location to spatial effect level which finally serves as the element of the temporal and spatial weighted matrix. As shown in Equation (2):

(2) $$STW_{\left\{\in l\right\}}=STW_{l,direct}+\left[STW_{l,spillover}diag\left(STW_{l,direct}\right)\right].*I_{Num_{\left\{\in l\right\}}}$$

$STW_{l,direct}$ is spatial and temporal weighted matrix of direct impact of sample area on target area.

$\left[STW_{l,spillover}diag\left(STW_{l,direct}\right)\right].\;\;*\;I_{Num_{\left\{\in l\right\}}}$ is spatial and temporal weighted matrix of indirect impact of sample area on target area. Among them, $STW_{l,spillover}$ is spatial and temporal weighted matrix of spillover effects of time and space in the sample area; $diag\;(STW_{l,direct}$) is the new vector formed by extracting the main diagonal elements from the matrix in brackets; $I_{Num_{\left\{\in l\right\}}}\;$ is the phase identity matrix of $Num_{\left\{\in l\right\}}$, the symbol.  ∗ denotes the dot product between matrix.

(1) The calculating method of indirect effect of spatial and temporal weighted matrix is shown in Equations (3)–(6)

(3) $$STW_{l,spillover}=TW_{l,spillover}⊗SW_{l,spillover}$$

In Equation (3), $TW_{l,spillover},\;\;SW_{l,spillover}$ are the initial spatial and temporal weight matrices of the spatial spillover effect relationship between two sample areas after standardization, the values of the matrix elements are from Equations (4) and (6), respectively, ⊗ is the Kronecker product.

(4) $$sw_{l,spillover}=\{\begin{array}{c} f\left(d_{n_{o}\to n_{d}},h_{d}\right),n_{o}\neq n_{d}\\ 0,n_{o}=n_{d} \end{array}$$

(5) $$f\left(.\right)=exp\left[-\frac{1}{2}\times ({\frac{d_{ij}}{h}}^{2}\right)]$$

In Equation (4), $sw_{l,spillover}$ is the element value of the initial spatial weight matrix of the sample region and the spatial weight matrix is formed after standardized processing to represent the spatial distance between the starting region $n_{o}$ and the destination region $n_{d};\;h_{d\;}$ represents the adaptive space bandwidth corresponding to the destination region $n_{d}$; in Equation (5), f(.) is the kernel function.

(6) $$tw_{l,spillover}\{\begin{array}{c} \frac{MI_{t_{d}}}{MI_{t_{o}}},t_{d}-t_{o}\\ 0,t_{d}-t_{o}<0 \end{array}$$

In Equation (6), $tw_{l,spillover}$ is the element value of the initial time weight matrix of the sample region and it is formed to $TW_{l,spillover}$ after a standardized processing, $t_{d},\;\;t_{o}$ represent the period numbers corresponding to the starting and destination regions, respectively, $MI_{t_{d}},\;\;MI_{t_{o}}$ represent the global Moran’s I calculated on the basis of all the region cross sections with the period numbers corresponding to the starting region and the destination region, respectively.

(2) The calculation of spatial and temporal weighted matrix of direct impact is shown in Equations (7) and (8).

(7) $$STW_{l,direct}=\left\{diag\left(TW_{l,spillover}\right).*I_{T_{\left\{\in l\right\}}}\right\}⊗{\mathrm{SW}}_{l,direct}$$

In Equation (7), $STW_{l,direct}$ is the spatial weight matrix of sample region’s direct spatial effect on target region, and the value of matrix element comes from Equation (8);

(8) $$sw_{l,direct}=f\left(d_{n\left\{\in l\right\}\to l,}h_{l}\right)$$

In Equation (8), $h_{l}$ is the adaptive spatial bandwidth corresponding to target region $l;\;d_{n\left\{\in l\right\}\to l}$ is the spatial distance between the area cross section n{∈l} and target region l. The kernel f(.) is shown in Equation (5).

(3) The bandwidth $h_{d}\;\mathrm{and}\;h_{l}$ are adjusted and the adjusted adaptive space bandwidth is finally obtained, which is shown in Equation (9).

(9) $$h=Max\left(d_{\left\{\in l\right\}\to l},d_{n_{o}\to n_{d}}\right)/\sqrt{-\frac{1}{n}ln\left(sevc\right)}$$

In Equation (9), the empirical constant n is 0.5, and the critical value of spatial effect is 0.05.

3. Based on the spatial and temporal weighted matrix and multiplication criterion, the data information of the sample region is mapped to the target analysis region so as to analyze the data information of the region, that is: $y_{l}\to STW_{\left\{\in l\right\}y\left\{\in l\right\}},X_{l}\to STW_{\left\{\in l\right\}}X_{\left\{\in l\right\}}$.

4. Finally, in combination with the Ordinary Least Square, the paper completed the parameter estimation process.

### 2.2. Construction of the Empirical Model

Based on modeling theory of STIRPAT model established by Dietz and Rosa [39], this paper expanded the STIRPAT model and conducted an empirical study by consulting the reference literature. 

The full name of STIRPAT is Stochastic Impacts by Regression on Population, Affluence, and Technology, and it was used to analyze the influence of the three independent variables of population, affluence, and technology on the dependent variable of environmental stress. Its basic model is demonstrated in Equation (10). When building the econometric model, the paper transformed the models by using the logarithms of both sides of the Equation (10), as shown in Equation (11):

(10) $$I_{i}=a*p_{i}^{b}*A_{i}^{c}*T_{i}^{d}*e_{i}$$

(11) $$LnI_{i}=a+b\left(lnp_{i}\right)+c\left(lnA_{i}\right)+d\left(lnA_{i}\right)+e_{i}$$

Equation (10) is the basic form of STIRPAT model in which I  stands for variable of environmental stress, p is variable of population, A is variable of affluence, T is variable of technology, i is individual of cross-section, a is a constant term, b,c,d represent, respectively, the coefficient of the three variables of population, affluence, technology, e is stochastic disturbance which meets the classic assumption.

This paper used carbon emission to represent the variable of environmental stress, and the original variables *b*, *c*, *d* are defined as population, economic development level, and energy intensity. From the perspective of the studies on influencing factors of carbon emissions, most scholars believe that population size, industrial structure, and foreign trade are important influencing factors of carbon emissions [40,41], so the above three factors are introduced into the model.

According to Equations (1) and (11), the empirical model of this paper is constructed in Equation (12)

(12) $$lnc_{\left\{i,t\right\}}=ln\beta_{0}\left(i,t\right)+\beta_{1}\left(i,t\right)lnX_{1,\left\{i,t\right\}}+\beta_{2}\left(i,t\right)lnX_{2,\left\{i,t\right\}}+\beta_{3}\left(i,t\right)lnX_{3,\left\{i,t\right\}}\;\;\;\;\;\;\;+\beta_{4}\left(i,t\right)lnX_{4,\left\{i,t\right\}}+\beta_{5}\left(i,t\right)lnX_{5,\left\{i,t\right\}}+\beta_{6}\left(i,t\right)lnX_{6,\left\{i,t\right\}}+\mu_{\left\{i,t\right\}}$$

In Equation (12), *C* stands for the total amount of regional carbon emissions, *X*_1_*, X*_2_*, X*_3_*, X*_4_*, X*_5_, and *X*_6_ are industrial structure, land area of urban construction, energy intensity, per capita GDP, population size, and foreign investment utilized, respectively. Definition and sources of relevant variables are shown in Table 1. Definition and sources of relevant variables.

### 2.3. Methods of Carbon Accounting

The level of carbon emission in a region can be generally typified by the carbon emission generated by the combustion of fossil energy [42]. Based on the guidance of IPCC [43], the paper calculated the carbon emissions in Beijing and Tianjin by using the coefficient of carbon emission [44]. As is shown in Equations (13) and (14).

(13) $$Qco2=\overset{10}{\underset{i=1}{\sum }}\;Ki\cdot Ei$$

(14) Ki=NCVi×CEFi×COFi×4412

Among them, Ei is the energy consumption of energy type i, which can be converted into standard coal according to some criterion. The coefficient *Ki* represents the specific net calorific value of energy type i. CEF is the content of carbon of each fossil fuel per calorific value and COF stands for carbon oxidation rate of each fossil fuel. The reference coefficient by which various energy is converted into standard coal and the emission coefficient of CO_2_ is shown in Table 2. Among them, the average low calorific value and conversion coefficient of standard coal are mainly derived from The General Rules for Calculation of Comprehensive Energy Consumption (GB/2589-2008); the content of carbon of each fossil fuel per calorific value and carbon oxidation rate are derived from The Preparation Guide for Provincial Greenhouse Gas List (Office of NDRC: Climate Volume 1041, 2011). Considering that most parts of China use coal-generated power and a few areas are based on hydropower, natural gas power, and wind power, and that CO_2_ generated by such clean energy as hydropower, wind power, and natural gas power can be omitted, the specific net calorific value consumed by electricity [45], specific net calorific value, carbon content per calorific value, and carbon oxidation rate consumed by electricity are therefore believed to be the same as those consumed by coal. 

Due to incomplete disclosure of energy consumption data of prefecture-level cities in Hebei Province, the above method cannot be used for carbon conversion. The paper borrowed Li ’s and Yang’s [46,47] principles of calculating the total amount of urban energy consumption (as is seen in Equations (15) and (16)), namely, the proportion of consumption of each energy in the total energy consumption of cities is the same as that of provinces. Assuming that the proportion of carbon emission from each energy consumption in carbon emission from the total of city is the same as that of the provincial level, the paper used Equations (16)–(18) to calculate the total carbon emission of each city. The specific calculation steps are as follows: Step 1: calculate the carbon emission of provincial energy type i (Equation (15)) Step 2: calculate the conversion coefficient of provincial carbon emission (Equation (16)) and take it as the conversion coefficient of municipal carbon emission (Equation (17)) Step 3: calculate the total carbon emission of each city (Equation (18)).

(15) $$Qco2_{i}=Ki\cdot Ei$$

(16) $$CEEI_{it}=\frac{PE_{it}+PG_{it}+PL_{it}}{CO_{it}+CK_{it}+PE_{it}+PG_{it}+PL_{it}+PT_{it}+KR_{it}+DS_{it}+FO_{it}+CQ_{it}}$$

(17) $$CEEI_{it}=CEEK_{it}$$

(18) $$COE_{it}=\frac{CE_{it}+CG_{it}+CL_{it}}{CEEK_{it}}$$

**Figure 2:**
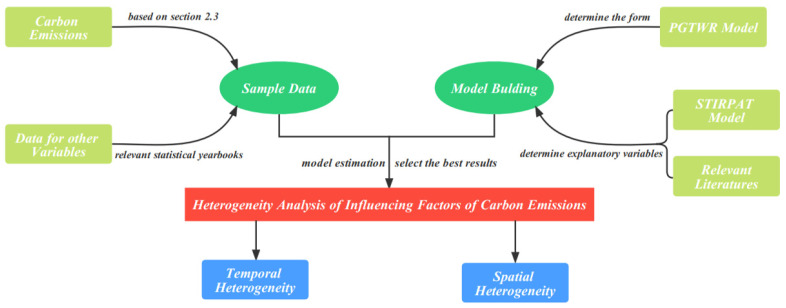
An overview of Materials and Methods.

In the equation, i is city, t is year, $Qco2_{i}$ represents the carbon emission from energy consumption of energy i, $CEEK_{it}$ stands for conversion coefficient of carbon emission of Province $\;i,\;COE_{it}$ stands for the total carbon emission of city i, $CO_{it}$ is coal consumption of Province i,$\;CK_{it}$ is coke consumption of Province i,$\;PT_{it}$ is petroleum consumption of Province i,$\;KR_{it}$ is kerosene consumption of Province i,$\;DS_{it}$ for diesel consumption of Province $i,\;FO_{it}$ is fuel oil consumption of Province $i,\;CQ_{it}$ is crude oil consumption of Province $i,\;CE_{it}$ is consumption of electricity of city i, $CG_{it}$ for consumption of natural gas of city $i,\;CL_{it}$ is consumption of liquefied petroleum gas of city i. Energy carbon emissions of each province are from China’s Energy Yearbook, and energy carbon emissions of each city are from China City Statistical Yearbook. An overview of Materials and Methods is shown below (Figure 2).

## 3. Results

### 3.1. PGTWR Estimation of Results

By using the standardized program complied in MATLABR 2020a by Fan and Guo [38], the paper estimated the regression equation of Equation (6), and the optimal spatial and temporal bandwidths based on AICc criteria were 13 and 6, respectively, which means that carbon emissions of a city in the Beijing–Tianjin–Hebei region are affected by the spatial influence from other 13 cities and the temporal influence of carbon emissions values of 6 years. The optimal spatial and temporal bandwidths based on the GCV criterion and RSS criterion are 7 and 6, respectively, which means that the spatial influence from the other seven cities and the temporal influence of the carbon emission values of 6 years have had an influence on the carbon emissions of one city in the Beijing–Tianjin–Hebei region. Since the selection result of optimal bandwidth based on the GCV criterion is basically equivalent in value to optimal bandwidth based on the CV criterion, the optimization of optimal spatial or temporal bandwidth based on CV criterion is not considered in this paper. The inconsistent optimal spatial and temporal bandwidths derived from AICc, GCC, and RSS criteria led to two different panel data which are produced by the effective neighboring local points included in the two optimal bandwidth dimensions. Consequently, based on the two optimal spatial and temporal bandwidths, respectively, this paper conducted an estimation on the PGTWR model, including the mixing effect, individual fixed effect, period fixed effect, and individual–period fixed effect. The result is shown in Table 3

Table 3 shows that F statistics estimated in the eight models all passed the hypothesis test of the significance level of 0.01, the goodness of fit was greater than 90%. Based on the significance ratio of the estimated value of local coefficient, CV, GCV, AIC, logarithmic likelihood value, and the estimate of variance of stochastic disturbance, the paper made a comprehensive evaluation of the result of models with the following judging criteria: the bigger the significance ratio of the estimate of local coefficient and the logarithmic likelihood ratio, the better; the smaller CV, GCV, AIC, and the estimate of the variance of stochastic disturbance, the better. 

**Figure 3:**
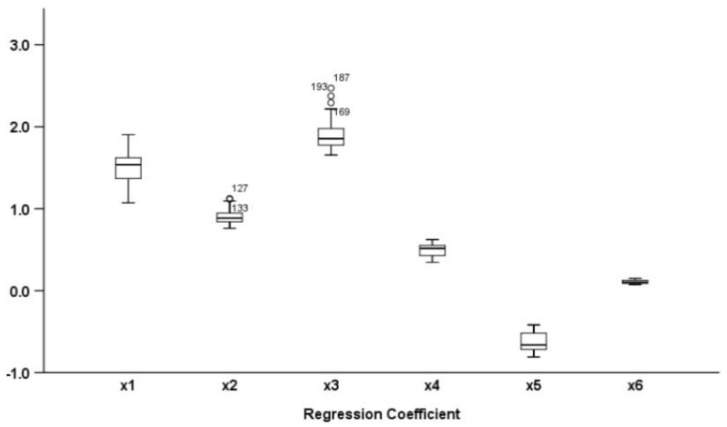
Box plot of regression coefficient of various explanatory variables of PGTWR model.

The significance ratio of the regression coefficients of the individual fixed effect and the individual–period double fixed effect under the two bandwidths were lower than 50%. The significance ratio of the regression coefficients of mixed effect and period fixed effect under bandwidth determined by GCV/RSS criterion is about 65%, and the significance ratio of the regression coefficients of period fixed effect and mixed effect under bandwidth determined by AICC criterion is 90%. After making further analysis and comparison of the statistical property of model results of the mixed effect and the period fixed effect under AICC criterion, the paper found (1) that significance ratio of the estimate of local coefficient of the period fixed effect was 0.9679, higher than 89.19% of the mixed effect, and (2) that estimated value of variance of stochastic disturbance, value of CV criterion, value of GCV criterion, and value of AICC criterion of the model as a whole were all lower than the mixed effect, and (3) that the logarithmic likelihood value is closer to 1 than the mixed effect. In conclusion, when the optimal spatial and temporal bandwidths were 13 and 6, respectively, the period fixed effect showed superior overall statistical properties. Table 4 and Figure 3 provide the descriptive statistics of the estimated results of PGTWR parameters from which we can see there are different degrees of variation in regression coefficients of each influencing factor of carbon emissions.

### 3.2. Temporal Heterogeneity Analysis of the Regression Coefficient of Influencing Factors of Carbon Emission

**Figure 4:**
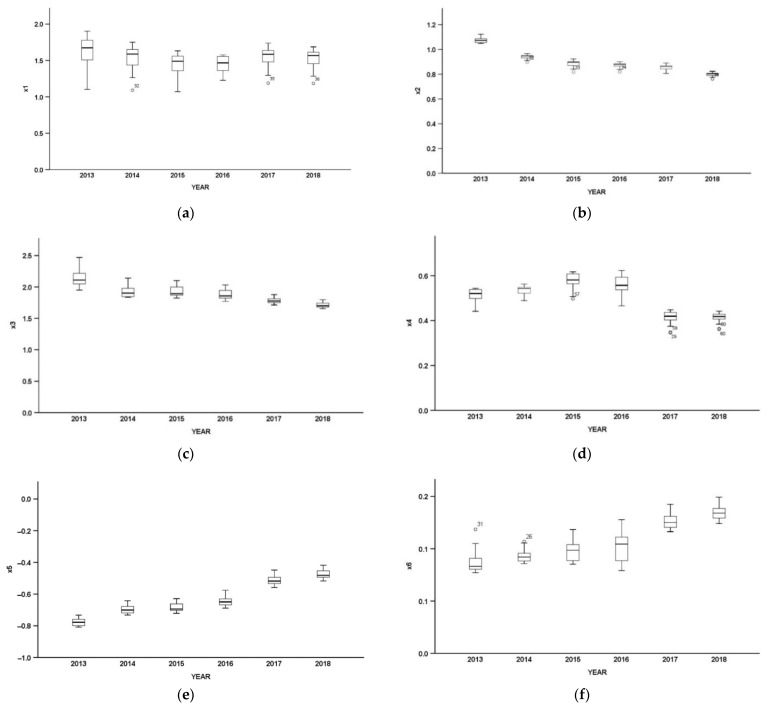
(**a**) Temporal heterogeneity of PGTWR regression coefficients of industrial structure; (**b**) Temporal heterogeneity of PGTWR regression coefficients of urbanization level; (**c**) Temporal heterogeneity of PGTWR regression coefficients of energy intensity; (**d**) Temporal heterogeneity of PGTWR regression coefficients of level of economic development; (**e**) Temporal heterogeneity of PGTWR regression coefficients of population size; (**f**) Temporal heterogeneity of PGTWR regression coefficients of opening up.

The paper did box plots of regression coefficient of various influencing factors of carbon emission in the cities of Beijing–Tianjin–Hebei according to years, respectively (Figure 4).

#### 3.2.1. Temporal Heterogeneity Analysis of the Influence of Industrial Structure on Carbon Emission

During the period of study, the industrial structure made a forward impact on carbon emissions. The degree of influence rose after falling first with a general downward trend, which is closely related to Beijing–Tianjin–Hebei Region’s efforts in elevating traditional manufacturing levels, promoting the added value of second industry products, strictly controlling the capacity of highly energy-consuming and high emission industries and developing low-carbon industries by means of high technology. The trend also suggests that the economy of Beijing–Tianjin–Hebei region is moving toward quality development. Meanwhile, the dispersion degree of the box plot tends to converge, indicating that the difference in economic development between the Beijing–Tianjin–Hebei region is gradually decreasing.

#### 3.2.2. Temporal Heterogeneity Analysis of the Impact of Urbanization Level on Carbon Emission

The direct and indirect demand of city life for energy is considered to be a major contributor to the adverse environmental impact of urbanization [48]. During the period of study, the influence of urbanization on carbon emissions in the Beijing–Tianjin–Hebei region decreased annually, mainly because the rise of urbanization rate and the population agglomeration in cities have promoted scientific and technological progress, facilitated the transition and upgrades of the industrial structure, and improved industrial efficiency through sharing, matching, and learning effect. At the same time, it has contributed to more intensive use of infrastructure, facilitated the agglomeration of economic activities and production behavior, and improved the efficiency of resources and energy use, thus effectively reducing the carbon emissions. In terms of policy, in recent years, China has vigorously implemented a new urbanization development strategy of economy and intensiveness, ecological and suitable living, and harmonious development, and advocated the idea of low-carbon living for urban residents as well. Therefore, the impact of the level of urbanization on carbon emission has gradually weakened.

#### 3.2.3. Temporal Heterogeneity Analysis of the Impact of Energy Intensity on Carbon Emission

During the study period, the influence of the energy intensity on carbon emissions has been relatively high, which is consistent with the fact that fossil energy is the main contributor to carbon emissions. The slow decline in the impact of energy intensity on carbon emissions indicates that the implementation of the strategy of Beijing–Tianjin–Hebei coordinated development has promoted intensive regional development as well as the progress of low-carbon technologies, thus improving the efficiency of energy utilization and reducing energy consumption per GDP. It also suggests that the economic structure of the Beijing–Tianjin–Hebei region has been constantly optimized and the proportion of tertiary industry has increased. The high economic benefits and low energy consumption of the tertiary industry have led to the decline in the influence of energy intensity on carbon emissions.

#### 3.2.4. Temporal Heterogeneity Analysis of the Impact of Level of Economic Development on Carbon Emission

During the study period, the overall impact of GDP on carbon emissions showed an inverted U-shape. From 2013 to 2015, the impact gradually increased, but it began to decline after 2016. In 2018, the impact was much lower than that in 2013, which shows that the economic aggregate increased the demand of various economic sectors for energy such as electricity and oil. Consumption of these fossil energies produced a great deal of carbon emissions. With the innovation of economic structure and improvement of the efficiency of input and output, the economy has gradually decoupled from carbon emissions, and GDP growth has mainly been fueled by consumption and scientific and technological innovation. In addition, the formulation of low-carbon economic strategy has controlled, and counteracted carbon emissions resulting from economic development. Therefore, the impact of economic development on carbon emissions has been decreasing.

#### 3.2.5. Temporal Heterogeneity Analysis of the Impact of Population Size on Carbon Emission

The regression coefficient between population size and carbon emission was a negative during the study period, mainly because population size has stimulated industry to grow rapidly and promoted the technological innovation and popularity of education as well as intensive development and application of energy-conserving technology. Meanwhile, population size contributes to providing personnel and technical support. The data reveal, however, that the advantage of carbon emission reduction brought by population size has been gradually disappearing.

#### 3.2.6. Temporal Heterogeneity Analysis of the Impact of Opening-Up on Carbon Emission

The influence of the level of opening-up on carbon emission was positive during the study period. With the deepening of opening up and the increase in utilization of foreign investments, building factories with investment and expanding the scale of pro duction will inevitably aggravate carbon emission. 

To sum up, the impact of industrial structure, urbanization level, and energy intensity on carbon emissions generally showed a downward trend, and the impact of population size and opening-up on carbon emissions showed an upward trend. The level of economic development increased first and then decreased. Population size had a negative impact on carbon emissions while the other five factors presented a stable positive impact, which was basically in line with the empirical expectation. The study also found that the status of some influencing factors was rising, while that of others was weakening. The overall numerical change has a certain guiding effect on policy making.

### 3.3. Spatial Heterogeneity Analysis of Regression Coefficient of Influencing Factors of Carbon Emission

**Figure 5:**
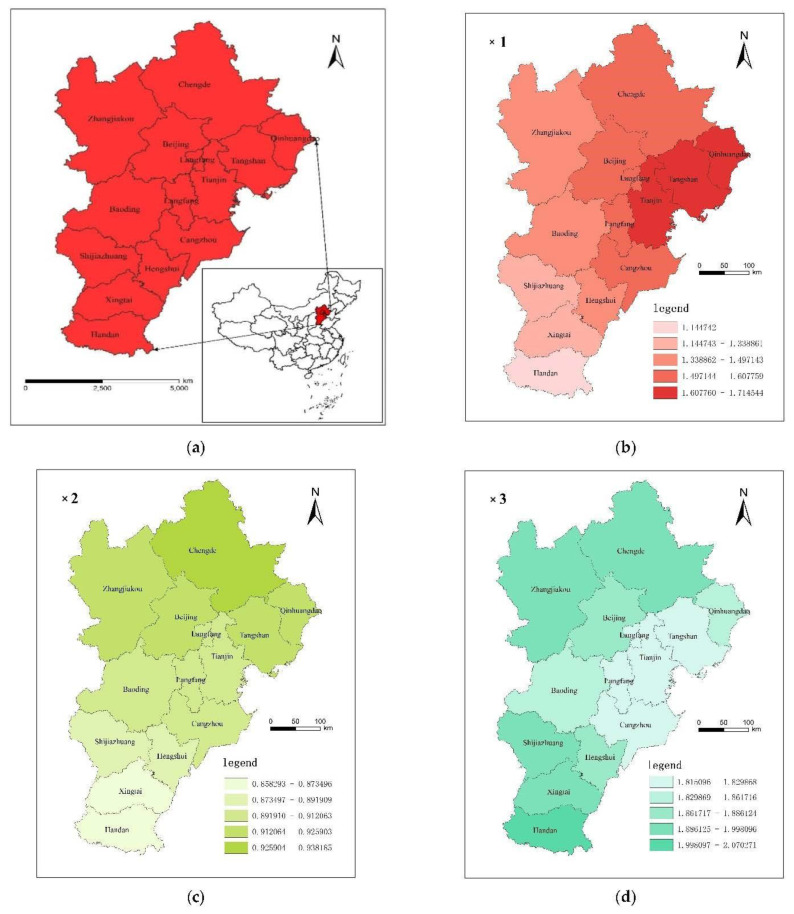

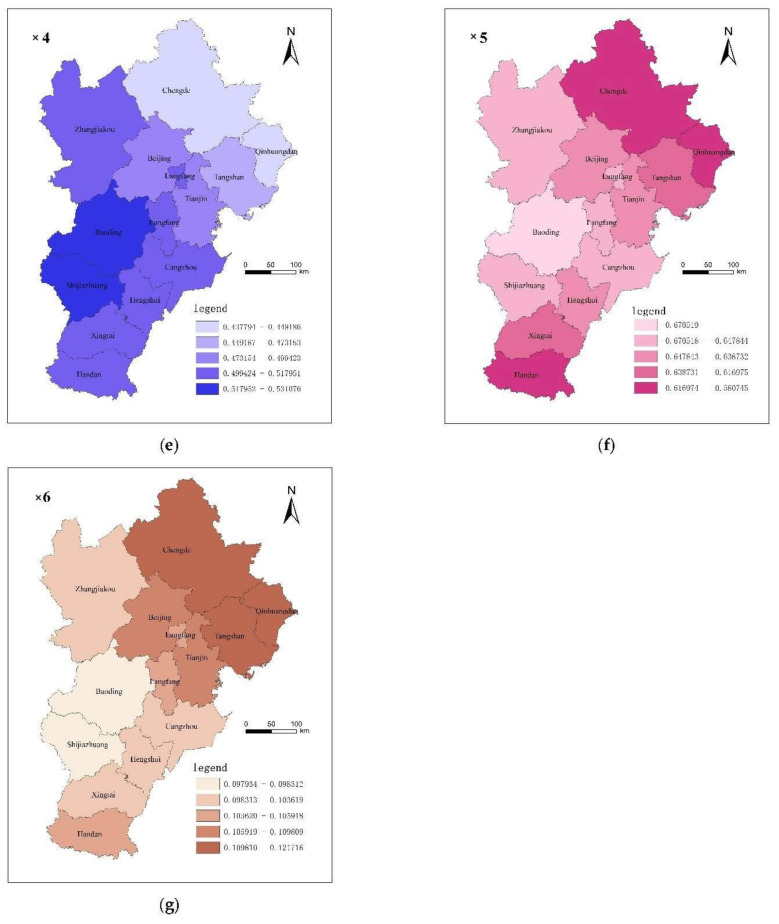
(**a**) Geographical location map of Beijing–Tianjin–Hebei region; (**b**) Spatial heterogeneity of PGTWR regression coefficient of industrial structure; (**c**) Spatial heterogeneity of PGTWR regression coefficients of urbanization level; (**d**) Spatial heterogeneity of PGTWR regression coefficients of energy intensity; (**e**) Spatial heterogeneity of PGTWR regression coefficients of level of economic development; (**f**) Spatial heterogeneity of PGTWR regression coefficients of population size; (**g**) Spatial heterogeneity of PGTWR.

To explore the spatial heterogeneity of regression coefficient of influencing factors of carbon emissions, the PGTWR model regression coefficients of influencing factors in indifferent regions and periods were averaged. With the use of ArcGIS10.2, the mean value was classified into five levels according to the rule of natural cutoff points and presented in the form of geographical map (Figure 5) so as to express more directly and analyze the spatial heterogeneity of regression coefficients.

The impact of industrial structure on carbon emission decreases from the east to the west. Tianjin, Tangshan, and Qinhuangdao are the most affected cities, followed by Cangzhou, Chengde, and Langfang, with Handan being the least influenced city. The influence of urbanization level on carbon emission weakens from the north to the south with Chengde being the most affected city, followed by Zhangjiakou, Beijing, Tangshan, and Qinhuangdao, and with Handan being the least influenced city. The influence of energy intensity on carbon emission declines progressively from the north–south to the middle regions, and Handan is the most influenced city, followed by Zhangjiakou, Chengde, Shijiazhuang, and Xingtai. The impact of economic development on carbon emission decreases from southwest to northeast, Shijiazhuang and Baoding are the most affected cities, and Chengde and Qinhuangdao are the least affected. The absolute value of regression coefficient of population size increases progressively from north–south to the middle regions, and Handan, Chengde, and Qinhuangdao have the highest absolute value. The lowest value is in Baoding. When it comes to the impact of the level of opening up on carbon emission, Qinhuangdao, Tangshan, and Chengde are the most affected cities, followed by Beijing and Tianjin. Among them, Qinhuangdao, Tangshan, and Tianjin are the major ports in the Beijing–Tianjin–Hebei region, among which Tianjin port is the largest port in north China, while Beijing, as the capital, has the capital airport with the most comprehensive functions, and thus enjoys the remarkable geographical advantage of aviation and a high level of opening up.

## 4. Conclusions and Policy Recommendations

### 4.1. Conclusions

This paper introduced the PGTWR model as the base model of the study, adopted thirteen cities as individuals of cross-section, and conducted a temporal and spatial heterogeneity study of the converted influencing factors of carbon emissions in Beijing–Tianjin–Hebei region with the time period from the year 2013 to 2018 as panel data. In terms of time and space as a whole, the regression coefficient of each influencing factor of carbon emission in Hebei Province has obvious heterogeneity. From the perspective of space, the differences in the impact of industrial structure and energy intensity on carbon emission vary significantly. As a result, these differences should attach importance when making the policy of carbon emission reduction. Relatively speaking, the heterogeneity of the influencing factors in Beijing–Tianjin–Hebei region is mainly reflected in the time dimension. In the period of study, the impact of industrial structure, the level of urbanization, energy intensity, and the level of economic development on carbon emission were on a declining curve while the impact of the level of opening up on carbon emission was on the rise, the regression coefficient of population size and carbon emissions had a negative impact on carbon emissions during the study period, but was gradually increasing and approaching zero point, and may change from negative to positive in the future, which indicates that the former four factors that reflect the level of economy and technology are not the focus of consideration when making the policy of carbon emission reduction, which is consistent with the conclusion that most cities in Beijing–Tianjin–Hebei region are in a strong decoupled status [49]. Therefore, more attention should be paid to the latter two factors for the time to come.

### 4.2. Policy Recommendations

The paper put forward the following recommendations. First, we should adjust the industrial structure and decrease the proportion of secondary industries. Among the cities in the Beijing–Tianjin–Hebei region, Tangshan’s secondary industry accounts for the highest proportion, with an average of nearly 60% during the study period. Its main industries have always been coal, oil, natural gas extraction, and other traditional fossil energy industries. The secondary industries of Cangzhou, Tianjin, and Qinhuangdao accounted for nearly 50%, also at a high level. Among the three industries, the secondary industry consumes mainly fossil energy and produces the most carbon emissions. Therefore, it is essential to accelerate the transformation of the economic development mode, lower the proportion of heavy industry, and develop the tertiary industry with low energy consumption and high output level. 

Second, energy intensity is one of the most important factors which affect carbon emissions. We should strengthen technological innovation, develop new energy technologies, eliminate high polluting and high energy consuming technologies, and increase the role of science and technology in empowering industrial development. We will concentrate on developing advanced manufacturing, new and high technology industries, and low-carbon and low energy-consuming industries so as to lower energy consumption per GDP and promote high-quality economic development. 

Third, we should steadily promote the development of urbanization, pay attention to ecological protection in the process of urbanization, and put an end to the expansion of urban scale by extensive development mode. This requires reasonable planning and layout according to the environmental self-purification capacity of different cities and towns as well as controlling the urban size below the ecological critical scale. Meanwhile, we should give play to the advantages of population scale to enhance infrastructure construction and keep improving the efficiency of resource allocation.

Fourth, while strengthening the introduction of foreign investment, we should pay attention to environmental protection, carry out environmental impact assessment on imported projects, and strictly control the introduction of projects with high carbon emissions. The Beijing–Tianjin–Hebei government should also absorb the successful experience of harmonious coexistence between foreign investment and environmental protection and formulate scientific and reasonable local policies so as to reduce the carbon emissions caused by foreign investment. 

Finally, the empirical results of the study not only make theoretical contributions to those literature of research on influencing factors and heterogeneity, but also make contributions to the formulation of carbon emission reduction policies in the Beijing–Tianjin–Hebei region of China. There are some shortcomings in this paper which point out the direction of further research. First of all, the paper takes the Beijing–Tianjin–Hebei region as its research sample, so the conclusion drawn is only applicable to the region, and the future studies can expand their research regions. Secondly, the paper takes the prefecture-level cities and above as its research sample, therefore, future studies can be conducted on the heterogeneity of county territory. Finally, the paper does not consider the influence of peripheral regions on the study region, so future studies can perform further research by introducing spatial lag term.

## Figures and Tables

**Table 1:** Definition and sources of relevant variables.

Variable	Variable Meaning	Unit	Data Source
Carbon emissions	CO_2_ emissions from fossil fuels	10,000 ton	See Section 2.3
Industrial structure	Proportion of output value of secondary industry	%	Local Statistical Yearbook
Urbanization level	Land area of urban construction	Square kilometers	China City Statistical Yearbook
Energy intensity	Energy consumption per unit of GDP	Tons of standard/100 million yuan	Local Statistical Yearbook
Level of economic development	GDP	One hundred million yuan	Chinese Statistical yearbook
Population size	Population of permanent residents	Ten thousand people	Local Statistical Yearbook
Opening up	Amount of foreign investment actually utilized	Thousands of dollars	Local Statistical Yearbook

**Table 2:** Calculation of CO_2_ emission coefficient.

Energy	Average Low Emission	Standard Coal Coefficient	Carbon Content Per Calorific Value	Carbon Oxidation Rate	CO_2_ Emission Coefficient
Coke	28,435 KJ/kg	0.7143 kgce/kg	26.37 tons of carbon/TJ	0.93%	1.9003 kg-CO_2_/kg
Natural gas of oil field	38.931 kg/m^3^	1.3300 kgce/m^3^	15.3 tons of carbon/TJ	0.99%	2.1622 kg-CO_2_/m^3^
Raw coal	20,908 KJ/kg	0.7143 kgce/kg	26.37 tons of carbon/TJ	0.94%	1.9003 kg-CO_2_/kg
Crude oil	41,816 KJ/kg	1.4286 kgce/kg	20.1 tons of carbon/TJ	0.98%	3.0202 kg-CO_2_/kg
Fuel oil	41,816 KJ/kg	1.4286 kgce/kg	21.1 tons of carbon/TJ	0.98%	3.1705 kg-CO_2_/kg
petroleum	43,070 KJ/kg	1.4714 kgce/kg	18.9 tons of carbon /TJ	0.98%	2.9251 kg-CO_2_/kg
kerosene	43,070 KJ/kg	1.4714 kgce/kg	19.5 tons of carbon/TJ	0.98%	3.0179 kg-CO_2_/kg
diesel	42,652 KJ/kg	1.4571 kgce/kg	20.2 tons of carbon/TJ	0.98%	3.0959 kg-CO_2_/kg
Liquefied petroleum gas	50,179 KJ/kg	1.7143 kgce/kg	17.2 tons of carbon/TJ	0.98%	3.1013 kg-CO_2_/kg

**Table 3:** Overall statistical properties of the example model under the two bandwidth dimensions and four kinds of effects.

	AICc Criterion (Optimal Spatial Bandwidth = 13, Optimal Temporal Bandwidth = 6)				GCV\RSS Criterion (Optimal Spatial Bandwidth = 7, Optimal Temporal Bandwidth = 6)			
	Mixing Effect	Individual Fixed Effect	Period Fixed Effect	Individual–Period Fixed Effect	Mixing Effect	Individual Fixed Effect	Period Fixed Effect	Individual–Period Fixed Effect
significance ratio of the estimate of local coefficient	0.8919	0.3397	0.9679	0.4423	0.6703	0.2885	0.6688	0.3376
Sample size	78	78	78	78	78	78	78	78
Degree of freedom	31	32	31	32	15	15	15	15
Estimate of Variance of stochastic disturbance	13.835	0.538	1.928	16.832	28.157	1.087	2.956	32.830
Value of CV criterion	428.9	17.2	59.8	538.6	422.4	16.3	44.3	492.4
Value of GCV criterion	0.0851	0.0034	0.0119	0.1069	0.0838	0.0032	0.0088	0.0977
Value of AICc criterion	448.1	192.2	291.7	461.2	501.7	245.2	323.5	511.3
Modified goodness of fit	0.9996	0.9814	0.9952	0.9999	0.9998	0.9319	0.9880	0.9999
F statistical value	48,378	1035	4521	3,822,745	80,040	264	1581	244,461
F probability of statistics	0.000	0.000	0.000	0.000	0.000	0.000	0.000	0.000
Modified critical value of probability (*α* = 0.01, 0.05, 0.1)	0.0169 0.0844 0.1688	0.0171 0.0853 0.1706	0.0202 0.1012 0.2024	0.0185 0.0924 0.1849	0.0149 0.0745 0.1489	0.0159 0.0796 0.1592	0.0178 0.0888 0.1775	0.0166 0.0829 0.1658
logarithmic likelihood values	−213.1	−86.5	−136.3	−220.8	−240.9	−113.9	−152.9	−246.8

**Table 4:** PGTWR model’s descriptive statistics of regression coefficient of various explanatory variables.

Variable	Minimum	Maximum	Average	Upper Quartile	Lower Quartile	Quartile Range	Standard Deviation
X1	1.07	1.90	1.51	1.38	1.62	0.23	0.18
X2	0.76	1.12	0.90	0.84	0.94	0.10	0.09
X3	1.65	2.47	1.90	1.78	1.98	0.19	0.16
X4	0.35	0.62	0.50	0.43	0.55	0.12	0.07
X5	−0.81	−0.42	−0.63	−0.71	−0.52	0.19	0.11
X6	0.08	0.15	0.11	0.09	0.13	0.04	0.02

## Data Availability

1. https://v.hbu.cn/https/77726476706e69737468656265737421f4f6559d6933665b774687a98c/yearbook/Single/N2019110026 (accessed on 6 March 2022); 2. https://v.hbu.cn/https/77726476706e69737468656265737421f4f6559d6933665b774687a98c/Yearbook/Single/N2020110103 (accessed on 6 March 2022); 3. https://v.hbu.cn/https/77726476706e69737468656265737421f4f6559d6933665b774687a98c/yearbook/Single/N2020020041 (accessed on 7 March 2022); 4. https://v.hbu.cn/https/77726476706e69737468656265737421f4f6559d6933665b774687a98c/yearbook/Single/N2020070178 (accessed on 7 March 2022); 5. https://v.hbu.cn/https/77726476706e69737468656265737421f4f6559d6933665b774687a98c/yearbook/Single/N2019110002 (accessed on 7 March 2022); 6. https://v.hbu.cn/https/77726476706e69737468656265737421f4f6559d6933665b774687a98c/yearbook/Single/N2020050229 (accessed on 7 March 2022); 7. https://v.hbu.cn/https/77726476706e69737468656265737421f4f6559d6933665b774687a98c/yearbook/Single/N2020120303 (accessed on 7 March 2022).
